# Supplementary figures and images for: Structural basis of Plasmodium vivax inhibition by antibodies binding to the circumsporozoite protein repeats
Source: eLife. 2022 Jan 13;11:e72908. doi: 10.7554/eLife.72908 (PMC8809896; doi:10.7554/eLife.72908)

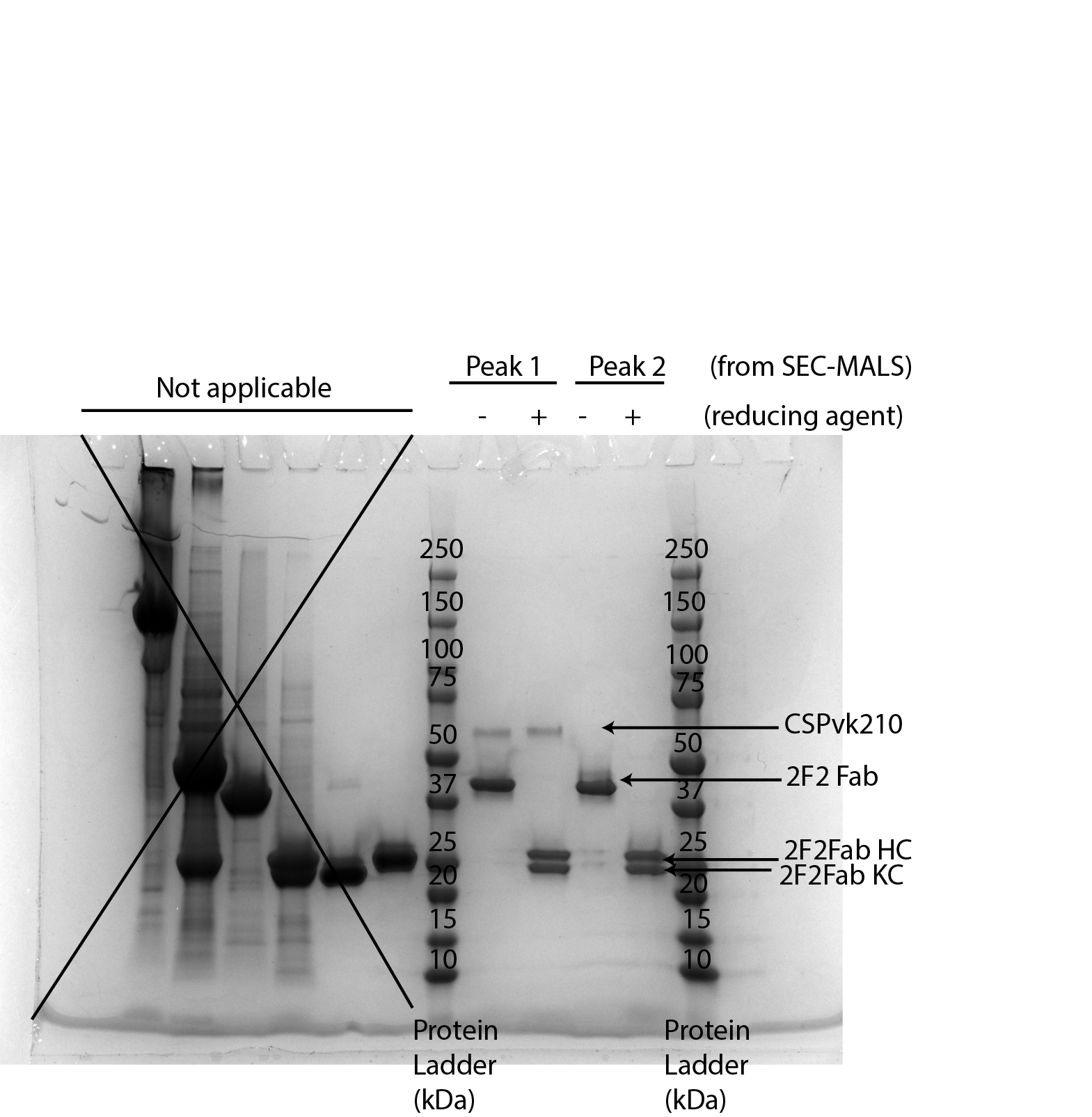

Supplement: Figure 6—source data 1. [file elife-72908-fig6-data1.zip › Gels Source data/Figure 6E source data 1 2F2 Fab PvCSP raw gel annotated.tiff]

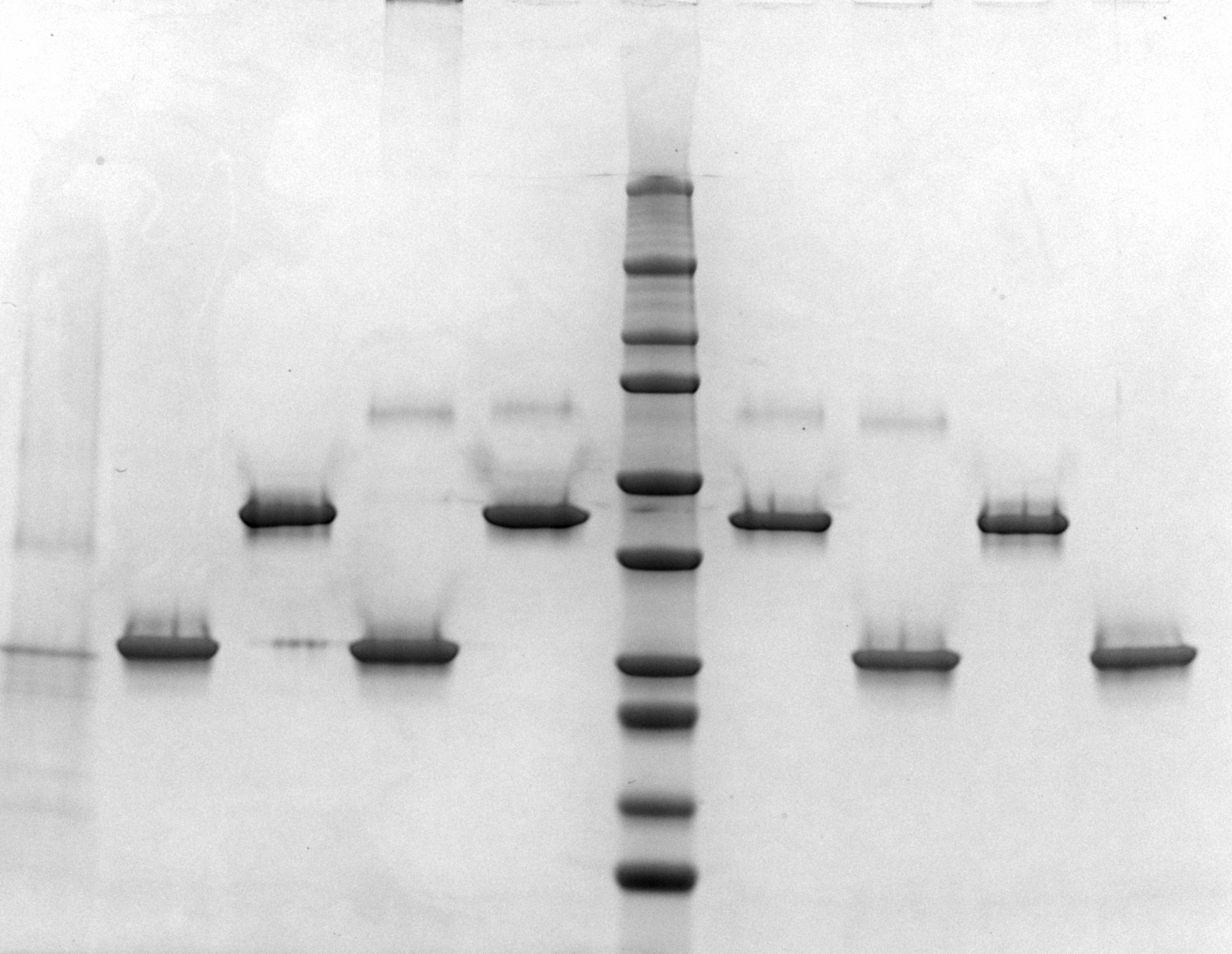

Supplement: Figure 6—source data 1. [file elife-72908-fig6-data1.zip › Gels Source data/Figure 6F source data 2 2E10E9 Fab PvCSP raw gel.jpeg]

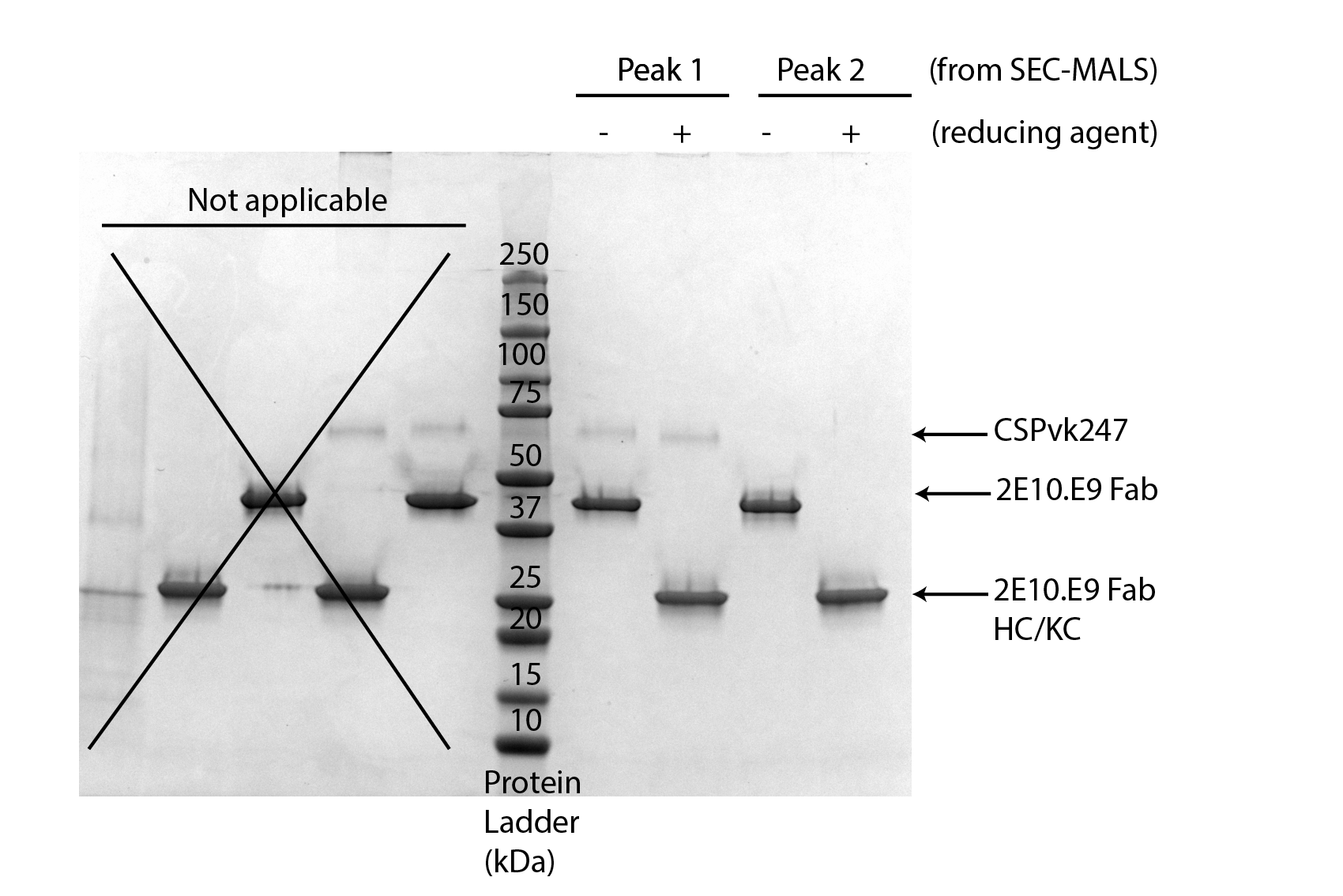

Supplement: Figure 6—source data 1. [file elife-72908-fig6-data1.zip › Gels Source data/Figure 6F source data 2 2E10E9 Fab PvCSP raw gel annotated.tiff]

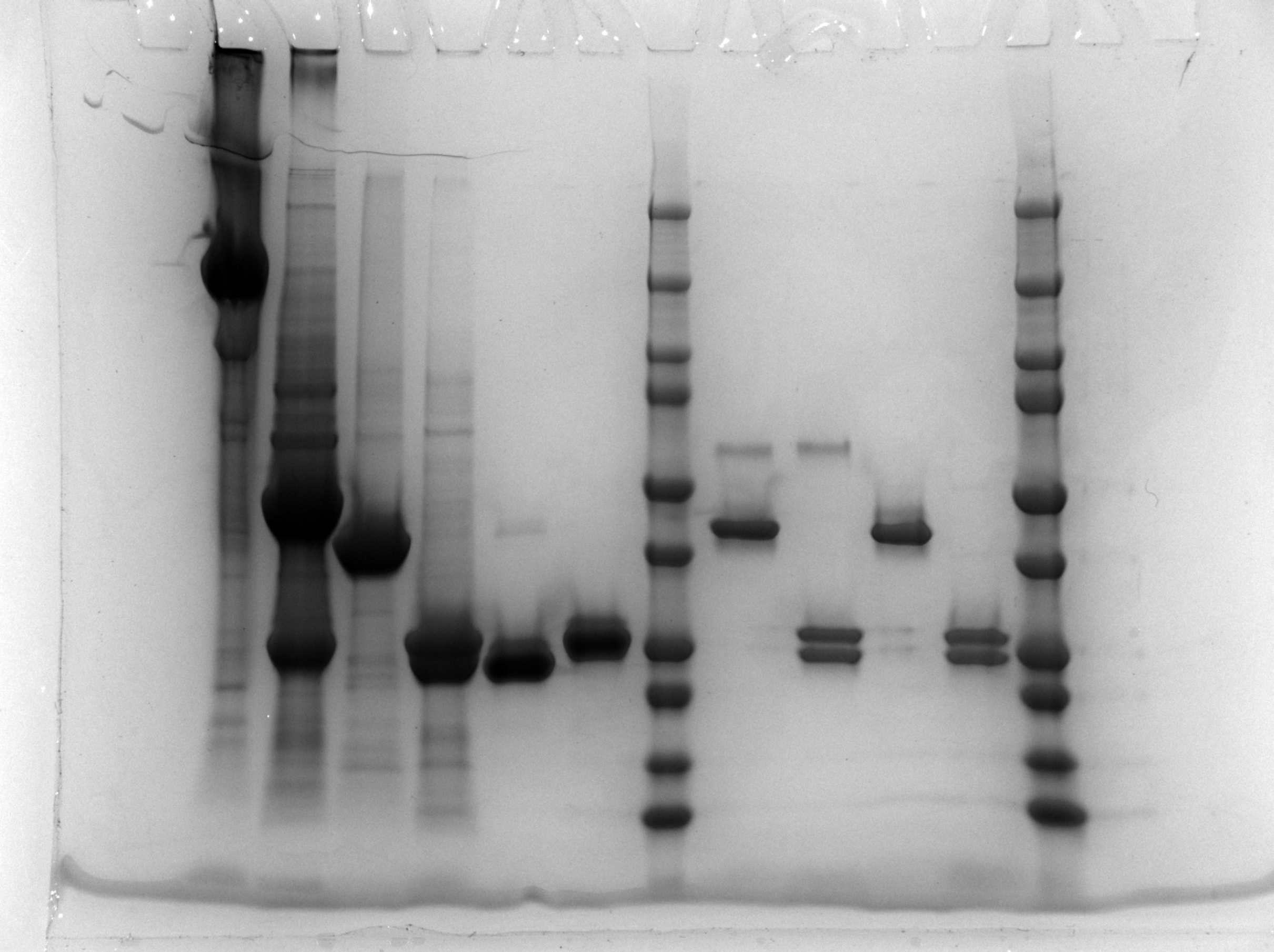

Supplement: Figure 6—source data 1. [file elife-72908-fig6-data1.zip › Gels Source data/Figure 6E source data 1 2F2 Fab PvCSP raw gel.png]
