# Supplementary material for: Structural basis of Plasmodium vivax inhibition by antibodies binding to the circumsporozoite protein repeats
Source: eLife. 2022 Jan 13;11:e72908. doi: 10.7554/eLife.72908 (PMC8809896; doi:10.7554/eLife.72908)
Supplement: Supplementary file 1. [file elife-72908-supp1.docx]

**Supplementary File 1**. Summary of CSP-derived peptides used in this study.

| Peptide | Origin | Sequence | Length (aa) | Analysis | | | | |
| --- | --- | --- | --- | --- | --- | --- | --- | --- |
|  |  |  |  | MD | CD | X-ray cryst. | ITC | NS EM |
| 210-1 | Pv VK210 | GDRADGQPAGDRADGQPA | 18 |  | ✓ | ✓ | ✓ |  |
| 210-2 | Pv VK210 | GDRAAGQPAGDRAAGQPA | 18 |  | ✓ | ✓ | ✓ |  |
| 210-3 | Pv VK210 | GDRADGQPAGDRAAGQPA | 18 |  | ✓ | ✓ | ✓ |  |
| 210-4 | Pv VK210 | GDRAAGQPAGDRADGQPA | 18 |  | ✓ | ✓ | ✓ |  |
| 210-5 | Pv VK210 | GDRAAGQPAGNGAGGQAA | 18 |  | ✓ | ✓ | ✓ |  |
| 210-6 | Pv VK210 | GDRADGQPAGDRADGQPAGDRADGQPA | 27 | ✓ |  |  |  |  |
| 210-7 | Pv VK210 | GDRAAGQPAGDRAAGQPAGDRAAGQPA | 27 | ✓ |  |  |  |  |
| 210-8 | Pv VK210 | GDRADGQPAGDRAAGQPAGDRADGQPA | 27 | ✓ |  |  |  |  |
| 210-9 | Pv VK210 | GDRAAGQPAGDRAAGQPAGNGAGGQAA | 27 | ✓ |  |  |  |  |
| 210-10 | Pv VK210 | GDRADGQPAGDRADGQPA  GDRADGQPAGDRADGQPA | 36 |  |  |  |  | ✓ |
| 247-1 | Pv VK247 | ANGAGNQPGANGAGNQPGANGAGNQPG | 27 | ✓ | ✓ |  | ✓ |  |
| 247-2 | Pv VK247 | EDGAGNQPGANGAGNQPGANGAGNQPG | 27 | ✓ | ✓ | ✓ | ✓ | ✓ |
| 247-3 | Pv VK247 | ANGAGNQPGANGAGNQPGANGAGGQAA | 27 | ✓ | ✓ | ✓ | ✓ |  |
| 247-4 | Pv VK247 | ANGAGNQPGANGAGNQPG | 18 |  |  | ✓ | ✓ |  |
| NPNDx2(1) | Pb ANKA | PPPPNPNDPPPPNPNDPPPPNPND | 24 |  |  |  |  | ✓ |
| NANP_5_ (2) | Pf NF54 | NANPNANPNANPNANPNANP | 20 |  |  |  |  | ✓ |

1. Kucharska I, Thai E, Srivastava A, Rubinstein JL, Pomès R, Julien J-P. Structural ordering of the Plasmodium berghei circumsporozoite protein repeats by inhibitory antibody 3D11. eLife. 2020 Nov 30;9:e59018.

2. Imkeller K, Scally SW, Bosch A, Martí GP, Costa G, Triller G, et al. Antihomotypic affinity maturation improves human B cell responses against a repetitive epitope. Science. 2018 Jun 22;360(6395):1358–62.
