# Supplementary material for: Structural basis of Plasmodium vivax inhibition by antibodies binding to the circumsporozoite protein repeats
Source: eLife. 2022 Jan 13;11:e72908. doi: 10.7554/eLife.72908 (PMC8809896; doi:10.7554/eLife.72908)
Supplement: Supplementary file 2. — No intramolecular H-bonds were detected for peptide 247-4. [file elife-72908-supp2.docx]

**Supplementary File 2**. Intramolecular H-bonds (3.0 Å cut-off) in PvCSP peptides observed in Fab-peptide co-crystal structures. No intramolecular H-bonds were detected for peptide 247-4.

| **210-1** | **210-2** | **210-3** | **210-4** | **210-5** | **247-2** | **247-3** |
| --- | --- | --- | --- | --- | --- | --- |
| Gln7^O^ – Gly10^N^ | Gln7^O^ – Gly10^N^ | Gln7^O^ – Gly10^N^ | Gln7^O^ – Gly10^N^ | Gln7^O^ – Gly10^N^ | Gly3^O^ – Gly5^N^ | Pro8^O^ – Ala13^N^ |
| Pro8^O^ – Asp11^N^ | Pro8^O^ – Asp11^N^ | Pro8^O^ – Asp11^N^ | Pro8^O^ – Asp11^N^ | Pro8^O^ – Asp11^N^ | Ala10^O^ – Gly12^N^ |  |
| Asp11^O^ – Asp14^N^ |  |  | Asp11^O^ – Asp14^N^ |  | Ala13^O^ – Gln16^N^ |  |
| Asp11^O^ – Gly15^N^ |  |  | Asp11^O^ – Gly15^N^ |  |  |  |
